# Long-Term and Short-Term Effects of Hemodialysis on Liver Function Evaluated Using the Galactose Single-Point Test

**DOI:** 10.1155/2014/260939

**Published:** 2014-07-10

**Authors:** Yi-Chou Hou, Wen-Chih Liu, Min-Tser Liao, Kuo-Cheng Lu, Lan Lo, Heng-Chih Pan, Chia-Chao Wu, Oliver Yoa-Pu Hu, Hung-Shang Tang

**Affiliations:** ^1^Department of Internal Medicine, Cardinal Tien Hospital, School of Medicine, Fu-Jen Catholic University, 362 Chung-Cheng Road, Hsin-Tien District, New Taipei City 23148, Taiwan; ^2^Department of Internal Medicine, Cardinal Tien Hospital, Yonghe Branch, New Taipei City 23445, Taiwan; ^3^Department of Pediatrics, Taoyuan Armed Forces General Hospital, Taoyuan 325, Taiwan; ^4^Division of Internal Medicine, Chang-Gang Memorial Hospital, Taoyuan, No. 5 Fusing Street, Gueishan, Taoyuan County 333, Taiwan; ^5^Department of Medicine, Tri-Service General Hospital, National Defense Medical Center, No. 161 Section 6 Minquan East Road, Neihu District, Taipei City 114, Taiwan; ^6^School of Pharmacy, National Defense Medical Center, No. 161 Section 6 Minquan East Road, Neihu District, Taipei City 114, Taiwan

## Abstract

*Aim*. The galactose single-point (GSP) test assesses functioning liver mass by measuring the galactose concentration in the blood 1 hour after its administration. The purpose of this study was to investigate the impact of hemodialysis (HD) on short-term and long-term liver function by use of GSP test.* Methods*. Seventy-four patients on maintenance HD (46 males and 28 females, 60.38 ± 11.86 years) with a mean time on HD of 60.77 ± 48.31 months were studied. The GSP values were compared in two groups: (1) before and after single session HD, and (2) after one year of maintenance HD.* Results*. Among the 74 HD patient, only the post-HD Cr levels and years on dialysis were significantly correlated with GSP values (*r* = 0.280, *P* < 0.05 and *r* = −0.240, *P* < 0.05, resp.). 14 of 74 patients were selected for GSP evaluation before and after a single HD session, and the hepatic clearance of galactose was similar (pre-HD 410 ± 254 g/mL, post-HD 439 ± 298 g/mL, *P* = 0.49). GSP values decreased from 420.20 ± 175.26 g/mL to 383.40 ± 153.97 g/mL after 1 year maintenance HD in other 15 patients (mean difference: 19.00 ± 37.66 g/mL, *P* < 0.05).* Conclusions*. Patients on maintenance HD for several years may experience improvement of their liver function. However, a single HD session does not affect liver function significantly as assessed by the GSP test. Since the metabolism of galactose is dependent on liver blood flow and hepatic functional mass, further studies are needed.

## 1. Introduction

Liver disease is a substantial contributor to the development of end-stage renal disease (ESRD). Patients with ESRD, especially those on hemodialysis (HD), are at high risk of hepatic viral infection. Traditional hepatic function tests including indicators for hepatocellular injury (aminotransferase levels), biliary tract injury (alkaline phosphatase, gamma glutamyl transpeptidase, and total bilirubin), and functional synthesis (albumin, urea, glucose, and prothrombin time) are routinely checked in patients on HD [1]. Nonetheless, none of these parameters indicate hepatic function satisfactorily. They may be normal even when active hepatic injury occurs in patients with azotemia or ESRD [2, 3] or be elevated falsely due to injury in other organs. Several studies demonstrated that patients with ESRD had elevated glutamic oxalacetic transaminase after HD, although the mechanism is unknown [2]. When considering hepatic clearing effects, hepatic clearance of sorbitol was similar before and after HD [4]. Thus, the effect of HD on actual residual liver function remains unclear.

Certain measurements are used to determine the amount of liver cells indirectly by measuring metabolic function or drug clearance as a substitute for quantitative liver function. The galactose single point (GSP) test is one novel method of assessing residual liver function. Galactose is a C-4 epimer of glucose and is catalyzed to glucose-1-phosphate by galactokinase, the rate-limiting step of metabolism for galactose exclusively in the liver [5, 6]. The ability of hepatocytes to metabolize galactose depends on total functioning liver mass and blood flow through the liver [7]. Galactose is catalyzed independent of cytochrome P450, so that its metabolism is less affected by drug induction or inhibition [8]. Recently, the US Department of Health and Human Services and the Food and Drug Administration approved the GSP test to determine the hepatic clearance of metabolized or nonmetabolized drugs [9, 10]. Hepatic clearance of cefoperazone by cirrhotic patients is correlated with GSP values [11]. GSP values also correlate with severity of cirrhosis and hepatocellular carcinoma [10]. The purpose of this study was to investigate the impact of HD on short- and long-term liver function as evaluated by the GSP test.

## 2. Methods

### 2.1. Subjects

Patients with ESRD (*n* = 74; 46 men and 28 women; mean age, 60.38 ± 11.86 yr) who underwent maintenance HD therapy at two nephrology units in different hospitals were prospectively investigated. All of the patients were informed about the study design and gave written consent to join the study. Patients were included in the study if they were more than 18 years of age, had stable, chronic renal failure (survival of ≥6 mo without active cardiovascular, cerebrovascular, hepatological, or infectious disease, cancer, pregnancy, or active hepatitis, liver cirrhosis or malignancy of the hepatobiliary system), and underwent maintenance HD three times per week. Patients with galactose intolerance (based on a questionnaire) or serious liver disease (based on a questionnaire, routine liver function tests, and liver sonography) were excluded from this study. The study protocol was in accordance with the principles of the Helsinki Declaration and was approved by the Human and Ethics Committees of the respective institutions.

All patients underwent conventional HD using a low-flux polysulfone dialyzer. The following baseline parameters were recorded on the day of blood sampling: body weight (post-dialysis weight), body mass index, mean blood pressure, hematocrit (Hct), fractional clearance of urea (*Kt*/*V*), normalized protein catabolic rate (nPCR), alanine transaminase (ALT), aspartate aminotransferase (AST), albumin, glucose, uric acid, and alkaline phosphatase (Alk-P). Serum urea levels were recorded pre- and postdialysis for the analysis of single-pool* Kt*/*V*, parameter of adequeacy for dialysis (Daugirdas, 1994). The nPCR, as the parameter of dietary protein intake, was calculated from monthly kinetic modeling sessions by applying the 2-blood urea nitrogen (BUN) method for the predialysis BUN level, and an estimate of the equilibrated postdialysis BUN level was obtained using the Daugirdas-Schniditz rate equation [12].

### 2.2. GSP Testing

GSP testing was performed after an 8-hour fast before each dialysis session. A galactose solution (0.4 g/mL) was infused intravenously at a dosage of 0.5 g/kg of body weight, and the infusion time was limited to 3 to 5 minutes. Blood sampling was performed 60 minutes after infusion. All 74 patients underwent predialysis GSP testing. Fourteen of the 74 patients were selected for evaluation of postdialysis GSP data after the HD session on the next week in order to avoid effect of residual galactose of pre-HD infusion. Postdialysis GSP was corrected according to postdialysis hematocrit. Another 15 of the 74 patients were selected for analysis of postdialysis GSP decreases after one year of maintenance HD.

### 2.3. Measurement of Galactose

Blood for each galactose test was withdrawn 60 minutes after galactose infusion; blood samples were obtained by venipuncture 60 min after injection. Predialysis GSP testing was performed before hemodialysis. Postdialysis GSP testing was done after HD was completed. During the HD session, each patient was in bed resting. Blood samples were kept in an ice bath until measured by an enzymatic method. A colorimetric galactose dehydrogenase (GADH) method was used to determine galactose levels using a modification of the neonatal screening test (Interscientific GAL570 nm, USA). The normal concentration range of the calibration curve was 50–1000 mg/mL. Same-day variation was evaluated by the standard deviation and percentage coefficient of variation (CV) for each concentration. A maximum 10% CV was permitted. The slope and intercept of the calibration curves were also checked on a daily basis [13].

### 2.4. Statistical Analysis

The sample size used in a study is determined based on Mead's resource equation. *E* = *N* − *B* − *T*, where *N* is the total number of individuals or units in the study (minus1), *B* is the blocking component representing environmental effects allowed for in the design (minus1), *T* is the treatment component corresponding to the number of treatment groups (including control group) being used or the number of questions being asked (minus1), and *E* is the degrees of freedom of the error component and should be somewhere between 10 and 20. In our study, the study using subjects is planned with two groups (pre-HD versus post-HD) (*T* = 1), with 15 numbers per group, making 30 subjects total (*N* = 29), without any further stratifications (*B* = 0); then, *E* would equal 28, which is above the cutoff of 20, indicating that our samples size may be large enough.

Data are presented as means ± standard deviations. We correlated predialysis GSP levels with baseline liver function and biochemical parameters using Pearson product-moment correlation. In selecting patients who received more than one GSP sampling, we used Student's paired *t*-tests to compare the differences in GSP levels before and after a single session of HD and after one year of maintenance HD. Statistical significance was defined as *P* < 0.05. All statistical analyses were conducted using SigmaStat for Windows, version 2.03 (SPSS Inc.; Chicago, IL).

## 3. Results

The geographic and demographic results of the 74 patients are shown in Table 1. Patients were on maintenance HD for a mean of 60.77 ± 48.31 months. The average patient age was 60.38 ± 11.86 years. The average galactose infusion time was 5.07 ± 1.69 minutes. The predialysis galactose level was 457.94 ± 297.83 g/mL. Six (8%) of the patients were hepatitis B carriers (positive for hepatitis B antigen) while four (5%) patients were hepatitis C carriers (positive for anti-hepatitis C antibody), which is lower than the carrier status of the general population in Taiwan [14].

**Figure 1:**
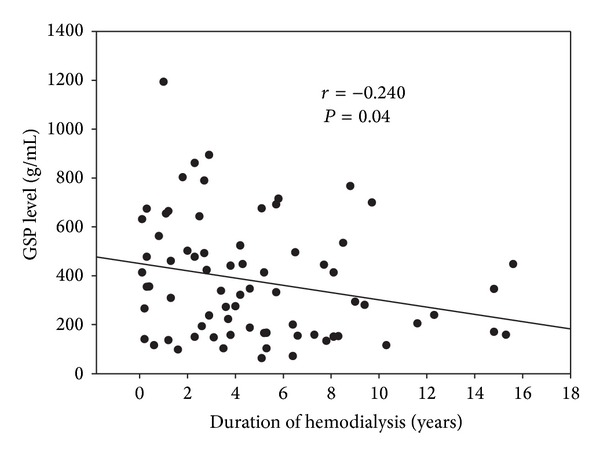
Significant negative correlation (*r* = −0.240, *P* = 0.04) between duration of hemodialysis of all patients and levels of galactose single point.

The correlations between GSP values and BUN, Cr, AST, ALT, albumin, uric acid, years on HD, and adequacy of dialysis (*Kt*/*V*) among the 74 HD patients are given in Table 2. Only the post-HD Cr level and years on dialysis correlated significantly with the GSP level (*r* = 0.280, *P* < 0.05 and *r* = −0.240, *P* < 0.05, resp.). Dialysis adequacy (*Kt*/*V*) had no correlation with GSP level.  Figure 1 also revealed a weakly negative correlation between duration of HD and the plasma GSP level (*r* = −0.240, *P* = 0.004).

**Figure 2:**
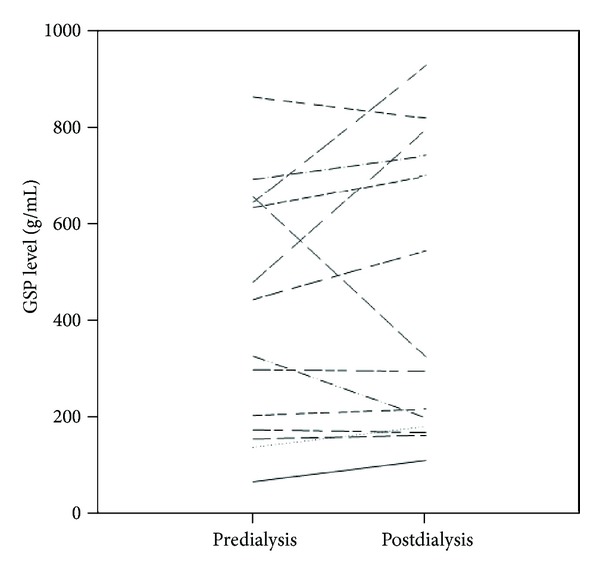
Of the 74 total patients, 14 were selected for comparison of galactose single-point (GSP) values (g/mL) before and after a single session of hemodialysis (HD). The average pre-HD and post-HD GSP values were 409.57 ± 256.12 g/mL and 438.86 ± 298.17 g/mL (corrected according to postdialysis hematocrit), respectively.

**Figure 3:**
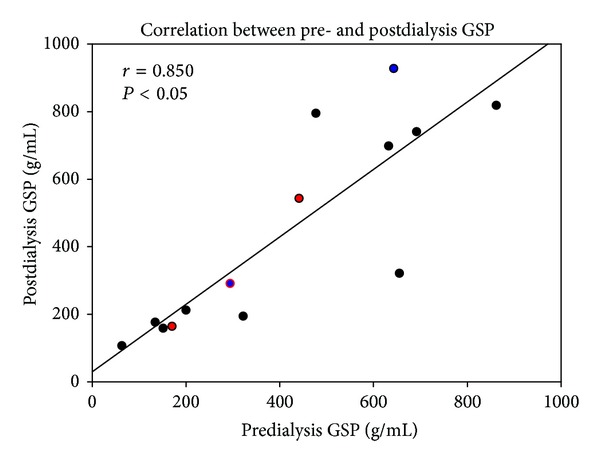
Significant positive correlation (*r* = 0.853, *P* < 0.05) between galactose single-point (GSP) values before and after a single hemodialysis session in 14 patients. Red circles: hepatitis B virus (HBV) carriers (*n* = 2); blue circles: hepatitis C virus (HCV) carriers (*n* = 2).

Figure 2 demonstrates the 14 patients who were randomly selected for GSP testing before and after a single session of hemodialysis. The average pre-HD and the average post-HD galactose, corrected by post-HD hematocrit, were 409.57 ± 256.12 g/mL and 438.86 ± 298.17 g/mL, respectively. Two of the 14 were HBV carriers and another two were HCV carriers. There are no statistic differences between pre- and postdialysis hepatic clearance of galactose (*P* = 0.49).  Figure 3 showed that pre-HD galactose levels correlated significantly with post-HD values (*r* = 0.851, *P* < 0.05).

**Figure 4:**
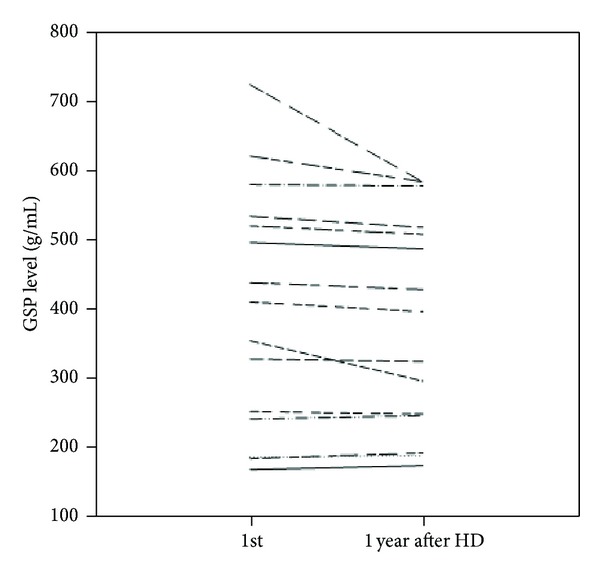
Differences in galactose single-point (GSP) test levels after 1 year of maintenance hemodialysis (HD) among 15 selected patients. The mean ± standard deviation (SD) of the first GSP value was 420.20 ± 175.26 g/mL. The 2nd GSP value after one year of maintenance HD was 383.40 ± 153.97 g/mL, with a difference of 19.00 ± 37.66 g/mL (*P* < 0.05).

Figure 4 shows the results of the 15 patients selected for analysis of GSP levels after 1 year of maintenance HD. These patients had no diabetes mellitus and their average age was 54.2 ± 8.3 years. The average years of maintenance dialysis of these patients were 4.5 ± 3.5 years. After one year of maintenance HD, pre-HD galactose levels decreased significantly from 420.20 ± 175.26 g/mL to 383.40 ± 153.97 g/mL (*P* < 0.05).

## 4. Discussion

We notice that one HD session did not result in changes in liver function compared to baseline as evaluated by GSP testing. Additionally, the galactose level seems to be correlated inversely with years of maintenance HD. Long-term HD of at least one year improved galactose metabolism (GSP testing). Hence, maintenance HD could have beneficial effects on liver function as assessed by GSP testing. We compared pre- and post-HD GSP levels in 14 patients after a single HD session, which did not affect GSP levels significantly. A single HD treatment did not have an immediate impact on hepatic metabolism of galactose. These patients had dialysis adequacy (*Kt*/*V*) greater than the National Kidney Foundation Kidney Disease Outcomes Quality Initiative guidelines [15] for low-flux dialysis, so uremic toxins of less than 1000 Da might not contribute to alteration of galactose metabolism by hepatocytes.

Hepatic blood flow also affects the metabolism of galactose. In rats, when blood flow was below a certain level, both galactose elimination and oxygen uptake were reversibly decreased in parallel with blood flow [16]. Cirrhotic dogs that underwent portocaval shunting or common bile duct ligation had decreased hepatic blood flow correlated with decreased galactose clearance [17]. Under conditions of low systemic blood flow or hemorrhage, perfusion of vital organs is maintained at the expense of perfusion of visceral organs, such as splanchnic ischemia [18]. Circulatory shock activates the sympathetic nervous system at the postcapillary mesenteric venules and results in autotransfusion to maintain cardiac performance [19]. However, total liver blood flow is somewhat protected when gut blood flow decreases, because hepatic arterial flow increases when portal venous flow decreases (the hepatic arterial buffer response). Adenosine, which induced hepatic arterial dilatation, is washed out under normal portal blood flow. When portal blood flow is compromised, adenosine accumulates in Mall's space so that hepatic blood flow is maintained [20]. Although few data for liver blood flow are available in renal failure, there are several human or animal studies about the effects of HD on hepatic blood flow. In uremic dogs undergoing dialysis, a decrease in circulating plasma volume through the ultrafiltration phase resulted in decreased cardiac output and was compensated by total peripheral resistance, which is impaired in the diffusion phase [21]. Leblanc et al. reported that, in chronic HD patients, although the hepatic clearance of sorbitol did not change significantly before and after HD, hepatic extraction of sorbitol, which inversely related to hepatic blood flow, decreased in comparison to healthy subjects [4]. Jakob et al. reported that patients who underwent hemodialysis had a decrease in splanchnic blood flow, cardiac output, and stroke volume under stable, traditional, clinical signs such as blood pressure [22]. All blood flows returned to baseline values after dialysis without therapeutic intervention, suggesting that this method of renal replacement therapy induced acute, but only temporary, reductions in splanchnic perfusion in intensive care patients with stable hemodynamics [22]. Additionally, patients under regular HD are forced to lie in bed 8–12 hours more than the general population every week. Hepatic arterial flow is higher in the supine position than in the sitting position, and drug metabolism may be influenced, especially among those with high first-pass metabolism [23]. Based on our results, HD improved hepatic blood flow and increase galactose metabolism.

We found that the patients who underwent maintenance HD have lower GSP level. HD affected hepatic metabolism of galactose in a chronic manner. Hepatocyte growth factor (HGF), a potent mitogen for hepatocytes, is released from peripheral blood mononuclear cells and mesenchymal cells after acute hepatic injury [24]. It accelerated hepatocyte regeneration and protected against toxic injury of hepatocytes in mice [25]. It also correlated with the severity of hepatic dysfunction in patients with fulminant hepatic failure [26]. Rampino et al. reported that HD was a potent stimulus of HGF production independent of heparin. In hepatitis C patients on chronic dialysis, mesenchymal cells, and peripheral blood mononuclear cells were stimulated by cytokines produced by leukocytes activated in the extracorporeal circulation. HGF released during dialysis is rapidly transformed into its biologically active form, and such an HGF increase is long lasting, up to 24 hours. Such endogenous HGF may prevent further hepatic injury and increase hepatocyte generation [27]. Borawski and Myśliwiec also found that high HGF levels were associated with the use of recombinant erythropoietin and unfractionated heparin [28]. Based on these reports, HFG is likely one of the factors contributing to improvement of galactose metabolism of patients in our study.

The correlation between galactose metabolism and hepatic blood flow in patients under HD is complicated. Studies in rats with glycerol-induced acute renal failure showed an initial decrease in cardiac output and liver blood flow during the first 12 hours after induction followed by an increase in 24 to 48 hours as measured using radioactive microspheres [29]. In end-stage renal failure, liver elimination of drugs is altered. Enzymatic activity may change as well as the hepatic extraction ratio. Recently, positron emission tomography (PET) was developed to use 2-18F-fluoro-2-deoxy-D-galactose for quantifying regional liver function [30]. Preoperative fluorodeoxyglucose PET also provides clinicians with the ability to predict liver reserve [31]. Therefore, a significant effort is still needed for linking correlation between blood flow and enzyme metabolism.

There are still several limitations in our study. Although we found that duration of hemodialysis may influence the GSP level, we could not get more case numbers because the procedure was time-consuming and galactose infusion might induce discomfort for some patients. Besides, since hepatic blood flow may influence the metabolism of galactose, further image studies such as Doppler scan on hepatic blood flow during hemodialysis or PET scan may be helpful. Since other cytokines such as HGF may be activated in HD patients, further studies for interaction between cytokines may help us understand the hepatic clearance in HD patients.

In conclusion, patients on maintenance HD for several years experience improvement of liver function. However, a single HD session does not appear to affect liver function significantly. Since the metabolism of galactose is dependent on liver blood flow and hepatic functional mass, whether the blood flow to the liver is slightly increased after HD, which may result in improved liver function as reflected in the GSP test, deserves further study.

## Figures and Tables

**Table 1:** The demographic data and biochemistry parameters of the patients in the study (*n* = 74).

Variables	Values	Value of reference
Demographics		
Age (years)	60.38 ± 11.86	
Female (%)	28 (37.84)	
Hepatitis B carrier (%)	6 (8.18)	
Hepatitis C carrier (%)	4 (5.40)	
Predialysis body weight (kg)	62.50 ± 9.89	
Etiology of end-stage renal disease		
Diabetes mellitus	14 (18.9%)	
Polycystic kidney disease	2 (2.7%)	
Preclampsia	1 (1.4%)	
Cormobidity		
Hypertension	16 (21.6%)	
Coronary artery disease	16 (21.6%)	
Malignancy	5 (6.75%)	
Medication		
ACEi/ARB	9 (12.1%)	
Statin	2 (2.7%)	
Phosphate binder	39 (52.7%)	
Acetaminophen	11 (14.8%)	
Phenytoin	2 (2.7%)	
Rifampicin	1 (1.4%)	
Biochemistry parameters		
Glucose (mg/dL)	124.83 ± 68.45	90–105
AST (U/L)	22.19 ± 17.04	<40
ALT (U/L)	18.47 ± 15.38	<40
Alk-p (U/L)	78.07 ± 56.28	30–95
Albumin (g/dL)	4.05 ± 0.35	3.5–5.0
Uric acid (mg/dL)	7.24 ± 1.19	4.0–6.2

Variables	Values	

Cr (mg/dL)		
Predialysis	11.42 ± 2.02	
Postdialysis	4.49 ± 3.32	
BUN (mg/dL)		
Predialysis	72.71 ± 21.51	
Postdialysis	19.13 ± 8.88	
Hematocrit (%)		
Predialysis	28.60 ± 5.60	
Post-dialysis	29.62 ± 6.26	
Months of Dialysis (months)	60.77 ± 48.23	
Pre-dialysis GSP (g/mL)	457.94 ± 297.83	

Note: *n* = valid cases, AST = Aspartate transaminase, ALT = alanine aminotransferase, Cr = serum creatinine, BUN = blood urea nitrogen, alk-p = alkaline phosphatase, GSP = galactose single point.

**Table 2:** Correlation between blood biochemistry parameters and GSP levels.

Parameters	GSP (*n* = 74)
AST	*r* = 0.125
ALT	*r* = 0.062
Glucose	*r* = 0.177
Alk-P	*r* = −0.005
Albumin	*r* = −0.073
Uric acid	*r* = 0.003
Cr (pre-HD)	*r* = 0.216
BUN (post-HD)	*r* = 0.031
Cr (post-HD)	*r* = 0.280∗
BUN (post-HD)	*r* = 0.156
Years on HD	*r* = −0.240∗
*Kt*/*V*	*r* = −0.124
nPCR	*r* = −0.086

Note: *n* = valid cases, AST = aspartate transaminase, ALT = alanine aminotransferase, Cr = serum creatinine, BUN = blood urea nitrogen, alk-p = alkaline phosphatase, and GSP = galactose single point. ∗*P* < 0.05.

## References

[1] Boone L, Meyer D, Cusick P (2005). Selection and interpretation of clinical pathology indicators of hepatic injury in preclinical studies. *Veterinary Clinical Pathology*.

[2] Cohen GA, Goffinet JA, Donabedian RK, Conn HO (1976). Observations on decreased serum glutamic oxalacetic transaminase (SGOT) activity in azotemic patients. *Annals of Internal Medicine*.

[3] Guh J, Lai Y, Yang C (1995). Impact of decreased serum transaminase levels on the evaluation of viral hepatitis in hemodialysis patients. *Nephron*.

[4] Leblanc M, Roy LF, Villeneuve J, Malo B, Pomier-Layrargues G, Legault L (1996). Liver blood flow in chronic hemodialysis patients. *Nephron*.

[5] Keiding S, Andreasen PB, Fauerholdt L (1973). Effect of phenobarbital induction on galactose elimination capacity in the rat. *Biochemical Pharmacology*.

[6] Keiding S, Johansen S, Winkler K, Tonnesen K, Tygstrup N (1976). Michaelis Menten kinetics of galactose elimination by the isolated perfused pig liver. *American Journal of Physiology*.

[7] Young TH, Tang HS, Chao YC (2008). Quantitative rat liver function test by galactose single point method. *Laboratory Animals*.

[8] Becker M (1998). 13C breath tests for measurement of liver function. *Gut*.

[9] Food and Drug Agency (2003). *Pharmacokinetics in Patients with Impaired Hepatic Function: Study Design, Data Analysis, and Impact on Dosing and Labeling, Guidance for Industry*.

[10] Tang H, Hu OY (1992). Assessment of liver function using a novel galactose single point method. *Digestion*.

[11] Yoa-Pu Hu O, Tang H-S, Chang C (1994). The influence of chronic lobular hepatitis on pharmacokinetics of cefoperazone novel galactose single-point method as a measure of residual liver function. *Biopharmaceutics and Drug Disposition*.

[12] Daugirdas JT, Schneditz D (1995). Overestimation of hemodialysis dose depends on dialysis efficiency by regional blood flow but not by conventional two pool urea kinetic analysis. *ASAIO Journal*.

[13] Hu OY-, Tang H-, Chang C- (1995). Novel galactose single point method as a measure of residual liver function: example of cefoperazone kinetics in patients with liver cirrhosis. *Journal of Clinical Pharmacology*.

[14] Centers for Disease Control (CDC)

[16] Keiding S, Vilstrup H, Hansen L (1980). Importance of flow and haematocrit for metabolic function of perfused rat liver. *Scandinavian Journal of Clinical & Laboratory Investigation*.

[17] Hanna SS (1984). Measurement of liver blood flow by galactose clearance. *Canadian Journal of Surgery*.

[18] Schlichtig R, Kramer DJ, Pinsky MR (1991). Flow redistribution during progressive hemorrhage is a determinant of critical O_2_ delivery. *Journal of Applied Physiology*.

[19] Reilly PM, Wilkins KB, Fuh KC, Haglund U, Bulkley GB (2001). The mesenteric hemodynamic response to circulatory shock: an overview. *Shock*.

[20] Lautt WW (1985). Mechanism and role of intrinsic regulation of hepatic arterial blood flow: hepatic arterial buffer response. *The American Journal of Physiology*.

[21] Kishimoto T, Sugimura K, Nakatani T (1983). The effects of diffusion and ultrafiltration on cardiac output and organ blood flows. *Proceedings of the European Dialysis and Transplant Association*.

[22] Jakob SM, Ruokonen E, Vuolteenaho O, Lampainen E, Takala J (2001). Splanchnic perfusion during hemodialysis: evidence for marginal tissue perfusion. *Critical Care Medicine*.

[23] Queckenberg C, Fuhr U (2009). Influence of posture on pharmacokinetics. *European Journal of Clinical Pharmacology*.

[24] Gallagher JT, Lyon M, Iozzo MV (2000). Molecular structure of heparan sulfate and interactions with growth factors and morphogens. *Proteoglycans: Structure, Biology And Molecular Interactions*.

[25] Shiota G, Wang TC, Nakamura T, Schmidt EV (1994). Hepatocyte growth factor in transgenic mice: effects on hepatocyte growth, liver regeneration and gene expression. *Hepatology*.

[26] Shiota G, Okano J, Kawasaki H, Kawamoto T, Nakamura T (1995). Serum hepatocyte growth factor levels in liver diseases: clinical implications. *Hepatology*.

[27] Rampino T, Libetta C, de Simone W (1998). Hemodialysis stimulates hepatocyte growth factor release. *Kidney International*.

[28] Borawski J, Myśliwiec M (2002). Serum hepatocyte growth factor is associated with viral hepatitis, cardiovascular disease, erythropoietin treatment, and type of heparin in haemodialysis patients. *Nephrology Dialysis Transplantation*.

[29] Hiley CR, Yates MS, Roberts PJ, Bloom AE (1980). Alterations in liver blood flow during glycerol-induced acute renal failure in the rat. *Nephron*.

[30] Sørensen M, Mikkelsen KS, Frisch K, Bass L, Bibby BM, Keiding S (2011). Hepatic galactose metabolism quantified in humans using 2-18F-fluoro-2-deoxy-D-galactose PET/CT. *Journal of Nuclear Medicine*.

[31] Ahn SG, Jeon TJ, Lee SD (2013). A survival benefit of major hepatectomy for hepatocellular carcinoma identified by preoperative [18F] fluorodeoxyglucose positron emission tomography in patients with well-preserved hepatic function. *European Journal of Surgical Oncology*.

